# Repurposing of statins for Buruli Ulcer treatment: antimicrobial activity against *Mycobacterium ulcerans*

**DOI:** 10.3389/fmicb.2023.1266261

**Published:** 2023-09-29

**Authors:** Juan Dominguez, Ana I. Mendes, Ana R. Pacheco, Maria J. Peixoto, Jorge Pedrosa, Alexandra G. Fraga

**Affiliations:** ^1^Life and Health Sciences Research Institute (ICVS), School of Medicine, University of Minho, Braga, Portugal; ^2^ICVS/3B’s - PT Government Associate Laboratory, Braga/Guimarães, Portugal

**Keywords:** *Mycobacterium ulcerans*, statins, drug repurposing, Buruli Ulcer, antimicrobial activity

## Abstract

*Mycobacterium ulcerans* causes Buruli Ulcer, a neglected infectious skin disease that typically progresses from an early non-ulcerative lesion to an ulcer with undermined edges. If not promptly treated, these lesions can lead to severe disfigurement and disability. The standard antibiotic regimen for Buruli Ulcer treatment has been oral rifampicin combined with intramuscular streptomycin administered daily for 8 weeks. However, there has been a recent shift toward replacing streptomycin with oral clarithromycin. Despite the advantages of this antibiotic regimen, it is limited by low compliance, associated side effects, and refractory efficacy for severe ulcerative lesions. Therefore, new drug candidates with a safer pharmacological spectrum and easier mode of administration are needed. Statins are lipid-lowering drugs broadly used for dyslipidemia treatment but have also been reported to have several pleiotropic effects, including antimicrobial activity against fungi, parasites, and bacteria. In the present study, we tested the susceptibility of *M. ulcerans* to several statins, namely atorvastatin, simvastatin, lovastatin and fluvastatin. Using broth microdilution assays and cultures of *M. ulcerans*-infected macrophages, we found that atorvastatin, simvastatin and fluvastatin had antimicrobial activity against *M. ulcerans*. Furthermore, when using the *in vitro* checkerboard assay, the combinatory additive effect of atorvastatin and fluvastatin with the standard antibiotics used for Buruli Ulcer treatment highlighted the potential of statins as adjuvant drugs. In conclusion, statins hold promise as potential treatment options for Buruli Ulcer. Further studies are necessary to validate their effectiveness and understand the mechanism of action of statins against *M. ulcerans*.

## 1. Introduction

*Mycobacterium ulcerans* is the causative agent of Buruli Ulcer, a neglected tropical skin disease characterized by necrotic lesions. Buruli Ulcer ranks as the third most common mycobacteriosis worldwide, exceeded only by tuberculosis and leprosy, and it is in poor rural areas of West and Central Africa that the highest incidence rates are reported, of which approximately 50% are children under the age of 15 (World Health Organization, 2023). In recent years, outbreaks of Buruli Ulcer have expanded geographically, with an alarming increase of reported cases in Australia (1.1 per 100,000 population in 2013 to 5.5 per 100,000 population in 2018) (Betts et al., 2020).

Buruli Ulcer pathogenesis is associated with mycolactone, an exotoxin produced by *M. ulcerans* that modulates the immune system, induces active cell death and analgesia (Guenin-Mace et al., 2019). Due to the indolent nature of Buruli Ulcer lesions and the absence of systemic symptoms, there is often a delay in healthcare seeking. Without treatment, non-ulcerative skin lesions can evolve into severe necrotic ulcers and spread to an entire limb (World Health Organization, 2023). The introduction of antibiotics in 2004 constituted a significant improvement of the therapeutic approach for Buruli Ulcer (World Health Organization, 2004; Etuaful et al., 2005; Chauty et al., 2007). Currently, the World Health Organization (WHO) recommends a combination based on rifampicin (10 mg/kg, orally once daily) and streptomycin (15 mg/kg, intramuscularly once daily) or clarithromycin (15 mg/kg, orally once daily) for a period of 8 weeks. This antibiotic regimen is highly effective for early clinical presentations and has drastically improved the recurrence rate (World Health Organization, 2012). Despite the efficacy of antibiotherapy, treatment of large ulcers still often requires additional extensive surgery and wound care in specialized hospitals (World Health Organization, 2012). Moreover, the treatment duration of 8 weeks, associated with the painful and potentially toxic daily injections of streptomycin, threaten patient compliance.

The development and commercialization of new drugs involves a huge investment in time and resources (Ashburn and Thor, 2004; Paul et al., 2010). Considering this scenario, drug repurposing has emerged as a viable option for the research of new treatments for infectious diseases. Drug repurposing is a practical and useful approach to exploit available and proven drugs outside the scope of the original medical indication for the development of new therapies. Moreover, drug repurposing has several advantages in terms of reduction of costs and time of drug implementation, due to the existence of clinical studies on safety, dosage, toxicity, and pharmacodynamics (Ashburn and Thor, 2004; Paul et al., 2010).

Throughout history, there have been many successful examples of drug repurposing. One potential candidate are statins. Statins are one of the most common drugs used to lower cholesterol production for the treatment of hypercholesterolemia, having a great impact on reducing the incidence of atherosclerosis and preventing cardiovascular events (Hennessy et al., 2016). In addition to its cholesterol-lowering properties, emerging reports have indicated several pleiotropic effects (Liao and Laufs, 2005), such as antimicrobial activity, including against mycobacteria and other bacteria, parasites, viruses and fungi (Lobato et al., 2014; Skerry et al., 2014; Rana et al., 2019). Following this reasoning, we tested the efficacy of different statins against *M. ulcerans* to determine their potential for the treatment of Buruli Ulcer.

## 2. Materials and methods

### 2.1. Bacteria

All work with *M. ulcerans* was carried out in Biosafety Level 3 (BSL3) Containment Facilities according to the Portuguese Legislation (Portaria n.° 1036/98). *M. ulcerans* strains 98–912, 94–1,327 and 00–1,441 are from the Institute of Tropical Medicine (ITM) collection in Antwerp, Belgium. The isolates were grown on oleic acid-albumin-dextrose complex (OADC)-supplemented Middlebrook 7H9 medium (BD Difco) with 1.5% agar at 32°C for approximately 4–6 weeks. For the preparation of the inoculum, *M. ulcerans* colonies were recovered, vortexed using 3 mm glass beads, diluted in phosphate-buffered saline (PBS), and the bacterial suspension was filtered through a 40-μm cell strainer to remove large aggregates.

### 2.2. Drugs

Synthetic lipophilic statins (atorvastatin, lovastatin, and fluvastatin, and simvastatin) and standard drugs for Buruli Ulcer treatment (rifampicin and streptomycin) were stored following the manufacturer’s instructions (Sigma-Aldrich). Rifampicin, atorvastatin, and lovastatin were dissolved in DMSO to a final 10 mg/mL for stock concentrations, while fluvastatin and streptomycin were dissolved in H_2_O at a concentration of 10 mg/mL and 1 mg/mL, respectively. Simvastatin was firstly activated by dissolving 4 mg of the drug in 100 μL of methanol, then 150 μL of 0.1 N NaOH was added, and incubated at 50°C in a water bath for 2 h. The pH was adjusted at 7.0 with HCl and final volume was completed up to 1 mL.

### 2.3. Drug susceptibility assay

The broth microdilution method for testing *in vitro* antibiotic activity for the above-mentioned drugs was used (Fraga et al., 2019). Briefly, 5×10^5^ CFU/well were cultured in OADC-supplemented Middlebrook 7H9 broth medium in a 96-well plate and incubated with a range of drug concentrations (for statins: 0.003052–400 μg/mL; for antibiotics: 0.000763–100 μg/mL) at 32°C for 7 days. After this period of incubation, 20 μL of resazurin 0.02% was added to each well and left for 2 more days for bacterial reduction of resazurin to resorufin. Bacterial growth inhibition was evaluated by measuring the optical density at 575/610 nm (Infinite 200 microplate reader, Tecan). Positive controls (bacteria without drugs) and negative controls (medium only) were used and considered as 100 and 0% of viability, respectively. We determined the IC50 that reflects the concentration at which a 50% inhibition occurs. IC50 provides a more conservative estimate of a drug’s inhibitory effect, which can be important when characterizing a drug’s potency, without excessive suppression of the target function and potential toxicity. The IC50 analyses were performed using GraphPad Prism 8 (GraphPad Software Inc., USA).

### 2.4. Cell culture

THP1 were obtained from the American Type Culture Collection (ATCC) and maintained in RPMI 1640 medium supplemented with 10% fetal bovine serum and 1% glutamine (Gibco, Thermo Fisher). THP1 monocytes were seeded at a density of 5×10^5^ cells/well and were differentiated to macrophages by exposing cells to 0.1 μM of phorbol 12-myristate 13-acetate (PMA) (Sigma-Aldrich) for 24 h. Macrophages were left to rest for 72 h before performing infection.

### 2.5. Macrophage infectivity assays and bacterial growth

Bacterial suspensions of *M. ulcerans* 98–912 were prepared as described above and further diluted in cRPMI before infecting naïve macrophage monolayers. *M. ulcerans* suspension (0.2 mL) was added to each well to achieve a multiplicity of infection (MOI) of 1:5 (bacteria to macrophage ratio) and incubated for 4 h at 32°C in a 5% CO_2_ atmosphere. Wells were then washed four times with warm cRPMI to remove noninternalized bacteria. Following infection, macrophages were incubated with cRPMI alone or supplemented with 2 μM atorvastatin, 2 μM simvastatin, 2 μM fluvastatin, and 1 μM rifampicin, for an additional 72 h, at 32°C in a 5% CO_2_ atmosphere. Statin concentrations were chosen based on those previously described by Lobato et al. (2014). After the incubation period, bacterial growth was assessed by CFU counting from macrophage monolayers. Briefly, macrophage monolayers were lysed with saponin (Sigma-Aldrich) (0.1% final concentration) and serial dilutions were plated on OADC-supplemented Middlebrook 7H9 medium with 1.5% agar at 32°C for approximately 4–6 weeks.

### 2.6. Isobologram analysis

Isobologram was constructed using a checkerboard method (Martinez-Irujo et al., 1996). The drug plates containing a two-dimensional array of rifampicin or streptomycin and atorvastatin or fluvastatin were prepared in a 96-well plate. The drugs were then incubated with 5×10^5^ CFU/well of *M. ulcerans* for 7 days at 32°C, after which 20 μL of resazurin 0.02% was added to each well and left for an additional 2 days for bacterial reduction of resazurin. The degree of drug interaction was determined using a fractional inhibitory concentration (FIC) method, which is calculated by the sum of the individual FIC values resultant from the division of each drug’s IC50 when used in combination by the IC50 of each drug when used alone. Synergy, additivity, and antagonism were classified based on the ΣFIC values, for which values ≤0.5 indicated a synergistic effect, values between 0.5–4 indicated an additive effect and values >4 indicated an antagonistic effect (Hall et al., 1983).

### 2.7. Statistical analysis

Data was expressed as mean ± standard deviation (SD) of at least three independent experiments. Normality was tested using Shapiro-Walk test, confirming normal distribution and variance homogeneity, followed by One-way analysis of variance (ANOVA) with Dunnett multiple comparisons test. Significance levels were set as *p < 0.05, ***p < 0.001, and ****p < 0.0001. Analyses were performed using GraphPad Prism 8.

## 3. Results

### 3.1. Statins have antimicrobial activity against *Mycobacterium ulcerans*

**Figure 1:**
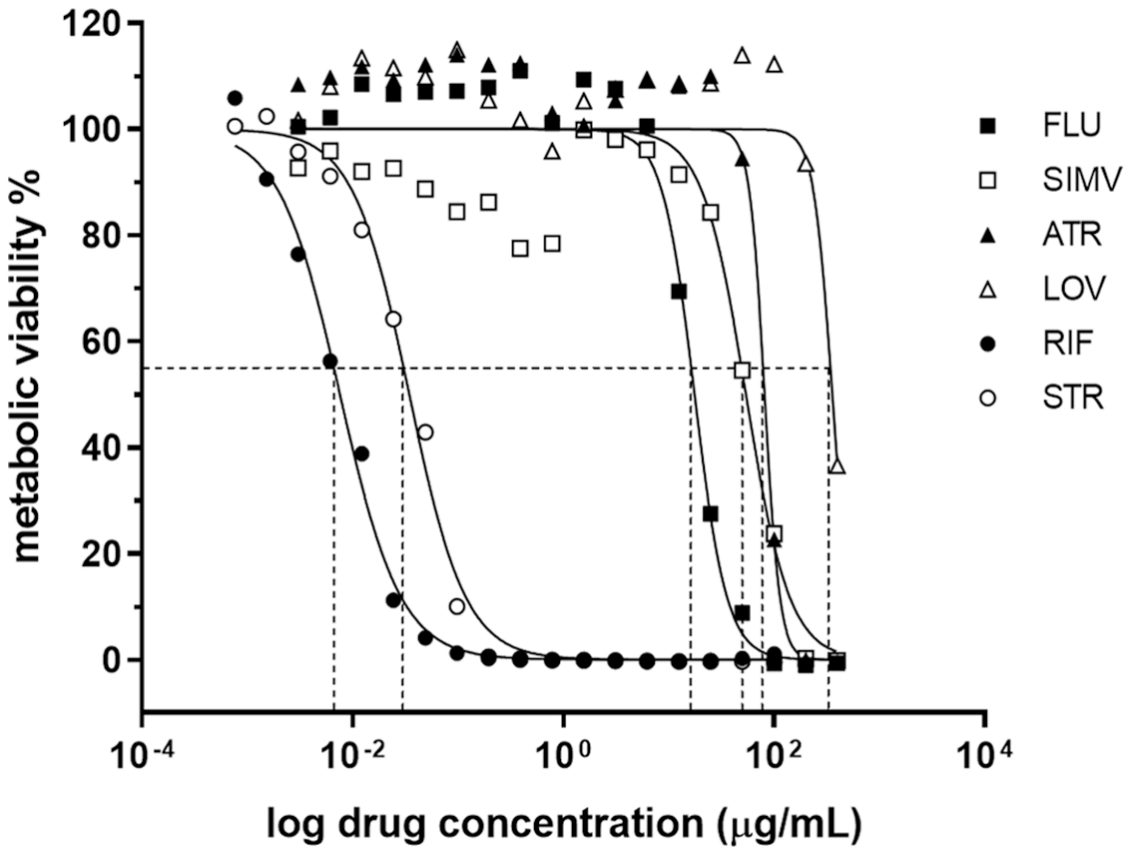
Dose–response curves for the antimicrobial activity of statins against *Mycobacterium ulcerans*. The broth microdilution assay was used to determine the dose–response curves of *M. ulcerans* against statins. *M. ulcerans* 98–912 was incubated for 7 days with increasing concentrations of statins [0.003052–400 μg/mL] (FLU, fluvastatin; ATR, atorvastatin; SIMV, simvastatin; LOV, lovastatin) or with standard antibiotics for BU treatment [0.000763–100 μg/mL] (RIF, rifampicin; STR, streptomycin). After this period of incubation, metabolic viability of *M. ulcerans* was determined with resazurin. Dashed lines represent the IC50 for each statin. Four independent experiments are represented and each experiment had three technical replicates.

Statins have been reported to present antimicrobial activity against different microrganisms, including bacteria, parasites, viruses, fungi and mycobacteria (Lobato et al., 2014; Skerry et al., 2014; Rana et al., 2019). To determine whether statins had an effect on *M. ulcerans* viability, drug susceptibility assays were carried out to obtain IC50 values of a panel of known statins, including fluvastatin, atorvastatin, simvastatin, and lovastatin, as well as of the standard antibiotics used for Buruli Ulcer treatment, rifampicin and streptomycin, as controls (Figure 1). As expected, rifampicin and streptomycin were effective at reducing mycobacterial activity, presenting IC50 values of 0.0076 μg/mL and 0.034 μg/mL, respectively. Importantly, increasing concentrations of different statins were also able to decrease *M. ulcerans* viability. Among the tested statins, fluvastatin had the highest inhibitory activity against *M. ulcerans* 98–912 (IC50: 17.60 μg/mL), followed by simvastatin (IC50: 54.13 μg/mL) and atorvastatin (IC50: 81.42 μg/mL; Table 1). Lovastatin was the statin with the highest, and most variable, IC50 value of 357.20 μg/mL. Due to the significant variability observed, lovastatin was excluded from the subsequent studies.

**Table 1:** Half-maximal inhibitory concentration (IC50) of statins against *Mycobacterium ulcerans*.

	Mean IC50 (μg/mL) (95% CI)
Rifampicin	0.0076 (0.005942–0.009656)
Streptomycin	0.034 (0.02759–0.04285)
Fluvastatin	17.60 (14.97–20.76)
Atorvastatin	81.42 (70.34–103.82)
Simvastatin	54.13 (41.74–69.7)
Lovastatin	357.20 (292–427.3)

**Figure 2:**
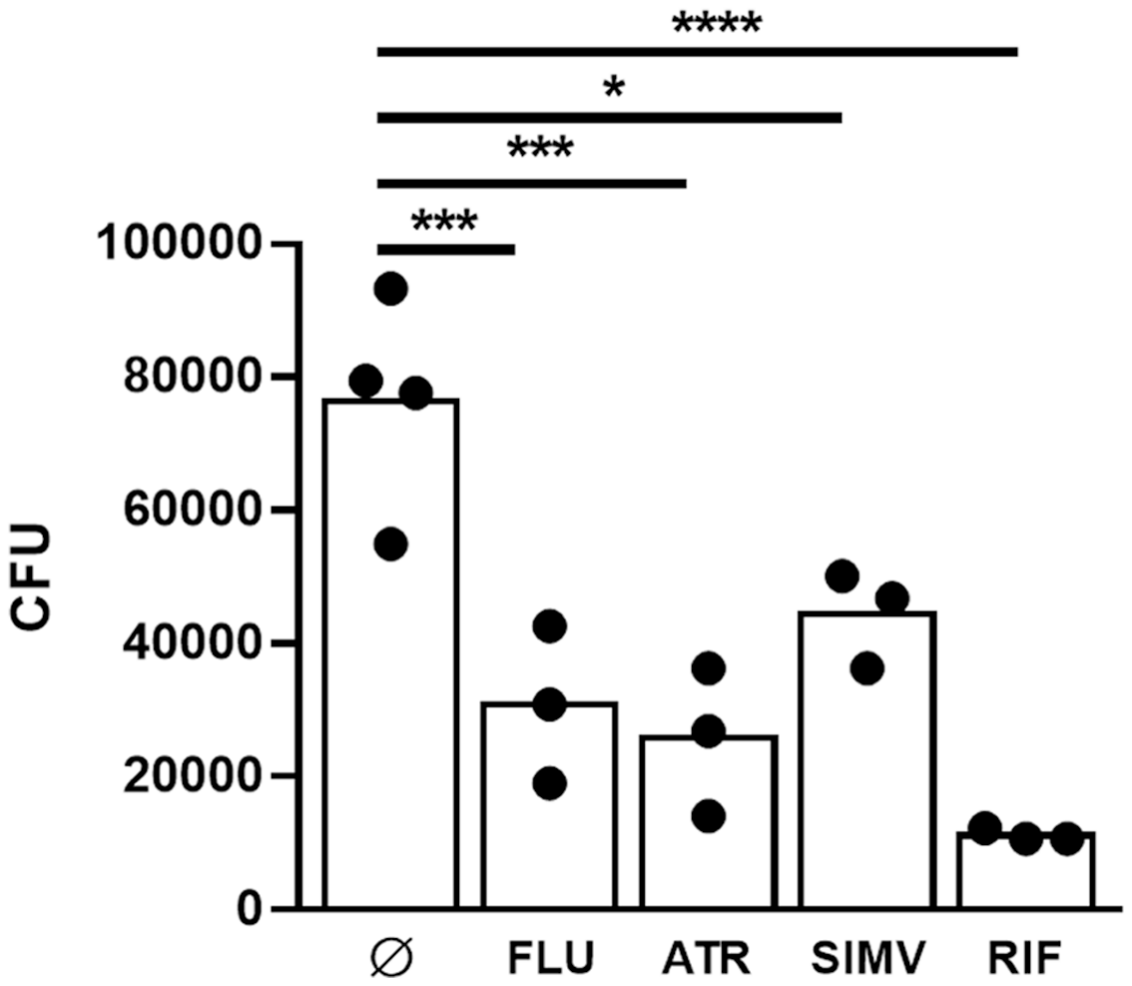
Statins have a mycobactericidal effect against *Mycobacterium ulcerans* infected macrophages. THP1 cells were differentiated to macrophages with PMA. *M. ulcerans* infected macrophages were treated with 2 μM ATR, 2 μM SIMV, 2 μM FLU, and 1 μM RIF for 72 h, after which CFU were performed. *p < 0.05; ***p < 0.001; ****p < 0.0001. One representative experiment of three independent experiments is shown. Each experiment had 3–4 technical replicates.

Importantly, the bactericidal effect associated to statin treatment was maintained, even in *M. ulcerans*-infected macrophages. Indeed, we observed a significant 2.5-3-fold CFU reduction, when in the presence of fluvastatin and atorvastatin, respectively, while simvastatin showed only a slight, albeit significant, 1.7-fold reduction in bacterial load (Figure 2). As expected, treatment of *M. ulcerans*-infected macrophages with rifampicin resulted in a 7-fold reduction of bacterial burden (Figure 2).

### 3.2. Statins have an additive effect upon combination with antibiotics against *Mycobacterium ulcerans*

Following confirmation of the antimicrobial activity of each individual statin against *M. ulcerans*, we next measured how *M. ulcerans* would respond to treatment when statins were used in combination with the recommended antibiotics for Buruli Ulcer treatment. For that, we carried out synergy testing to assess whether the *in vitro* interaction of the statin-antibiotic combination would act (i) additively, where the cumulative effect would be the sum of the two antimicrobials acting independently; (ii) synergistically, where the combined activity of the drugs is greater than the sum of their effects when used individually or; (iii) antagonistically, where the combined effect of the drugs is less than the sum of each drug when used individually (Doern, 2014).

Several synergy testing methods are available (Doern, 2014), but considering the slow growth rate of *M. ulcerans* (around 4–6 weeks to grow in culture), the checkerboard method was chosen to assess the cumulative efficacy of statin-antibiotic combinations against *M. ulcerans*. Antimicrobial combinations that result in FIC ≤ 0.5 are synergistic. FIC values between 0.5 to 4.0 are considered to represent an additive effect, while those above 4 represent an antagonistic effect (Hall et al., 1983). The FIC values are then further used to perform an isobologram analysis to evaluate statin-drug interaction.

**Figure 3:**
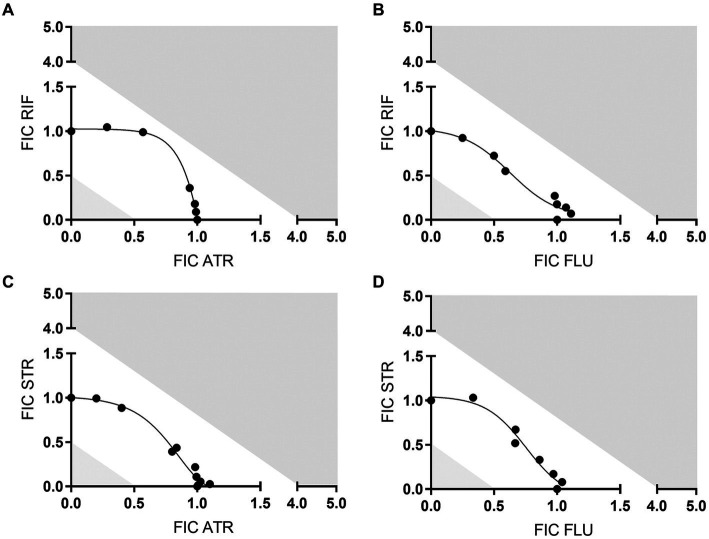
Isobologram analysis between statins and antibiotics. The plots show the fractional inhibitory concentration (FIC) values represented the drug interaction between rifampicin (RIF) or streptomycin (STR) and atorvastatin (ATR) or fluvastatin (FLU) against *M. ulcerans*: **(A)** FIC RIF vs FIC ATR; **(B)** FIC RIF vs FIC FLU; **(C)** FIC STR vs FIC ATR; **(D)** FIC STR vs FIC FLU. Antimicrobial combinations that result in FIC values ≤0.5 are synergistic; FIC values between 0.5 and 4 are additive; FIC values > above 4 are antagonistic. Shaded areas correspond to the above-mentioned cut-offs. One representative experiment of three independent experiments is shown. Each experiment had three technical replicates.

Among the statins that were tested, atorvastatin and fluvastatin presented the most evident antimycobacterial effects (Figures 1, 2). As a result, these two statins were selected for further investigation regarding their potential interactions in combination with rifampicin or streptomycin. Isobologram analysis of the different combinations between the drugs showed FIC values between 1 and 1.5, indicating an additive effect for the combination atorvastatin and rifampicin or streptomycin, as well as fluvastatin and rifampicin or streptomycin (Figure 3).

## 4. Discussion

The implementation of antibiotic regimens for the treatment of Buruli Ulcer was a significant breakthrough, not only by being highly effective for early clinical presentations, but also by enhancing the recovery of severe cases avoiding surgical intervention (World Health Organization, 2004, 2012; Chauty et al., 2007). Nevertheless, antibiotic treatment still faced limitations associated with the daily intramuscular streptomycin injection (World Health Organization, 2004; Chauty et al., 2007); low compliance due to prolonged treatment time (World Health Organization, 2004; Klis et al., 2014); streptomycin-associated adverse effects ranging from hearing loss to nephrotoxicity (Klis et al., 2014; Phillips et al., 2020); the gradual increase of antimicrobial resistance (Owusu et al., 2016); the development of paradoxical reactions characterized by an increase in lesion size after an apparently successful antibiotic treatment (Frimpong et al., 2019); and the refractory efficacy of antibiotic treatment for large lesions with extensive necrosis and avascular tissue (Pommelet et al., 2014). Collectively, these limitations prompted the investigation of new therapeutic interventions for Buruli Ulcer. Efforts have been directed at replacing streptomycin with alternative antibiotics, such as clarithromycin. Recently, a randomized, open-label, phase 3 trial showed non-inferiority effectiveness between an oral clarithromycin/streptomycin 8-week regimen in comparison with the standard therapy (Phillips et al., 2020). Moreover, in countries such as Japan and Australia, oral therapies including rifampicin, clarithromycin and fluoroquinolones are part of the common practice (O'Brien et al., 2014; Sugawara et al., 2015; Johnson, 2020).

The search for suitable alternative drugs for the treatment of infectious diseases has expanded beyond traditional antibiotics, including drug repurposing, a convenient and economical approach to exploit available drugs outside the scope of their original medical indication, allowing their timely implementation in limited resource countries.

Statins are one of the most used drugs worldwide with around 40 million of users. Their general chemical structure is characterized by the presence of a conserved lactone ring or substituents, but slight differences in chemical structure confer them individual lipophilic/hydrophilic properties that ultimately impact their level of absorption, distribution, metabolic action, and excretion (Corsini et al., 1999; Schachter, 2005). Indeed, lipophilic statins, which include atorvastatin, simvastatin, fluvastatin, lovastatin, and pitavastatin, easily cross cell membranes by passive diffusion and therefore achieve widespread distribution across tissues. In contrast, hydrophilic statins, namely rosuvastatin and pravastatin, rely on protein transporters found on the polar cell membrane for uptake in the liver, potentially limiting their effectiveness beyond this organ (Climent et al., 2021). Statins are structural analogs of mevalonate that competitively bind to the active site of 3-hydroxy-3-methylglutaryl-Coenzyme A (HMG-CoA) reductase, a rate-limiting enzyme involved in cholesterol biosynthesis. HMG-CoA reductase is required not only for cholesterol synthesis but also feeds isoprenoid metabolism by providing isoprenoid precursors, such as isopentenylallyl diphosphate (IPP) and dimethylallyl diphosphate (DMAPP). These drugs are mainly used for the treatment of hypercholesterolemia, having a great impact in reducing the incidence of atherosclerosis and preventing cardiovascular events (Endo, 2010). Emerging reports have also indicated several pleiotropic effects, including antioxidant, anti-inflammatory, anti-thrombotic, immunomodulatory, and plaque-stabilizing properties (Liao and Laufs, 2005). Importantly, various articles have also reported antimicrobial effects of statins against various types of microorganisms, including mycobacteria (Lobato et al., 2014; Gupta and Kumar, 2019).

By using the well-established broth microdilution assay, we identified atorvastatin, fluvastatin and simvastatin as being effective at reducing *M. ulcerans* viability, while lovastatin required a much higher concentration to inhibit bacterial growth. These results hold true regardless the origin of the *M. ulcerans* strain and the type of mycolactone produced, at least for atorvastatin and fluvastatin (Supplementary Figure S1). Although the exact mechanisms underlying statin’s direct antimicrobial effect are not known, it is unlikely associated to the inhibition of bacterial HMG-CoA reductase. Indeed, amino acid sequence analysis has suggested the existence of two distinct classes of HMG-CoA reductase: class I HMG-CoA reductases, present in all eukaryotes, and class II forms of the enzyme, present in some eubacteria (Bochar et al., 1999), the latter presenting a significantly weaker affinity for statins than the enzyme found in eukaryotes (Bischoff and Rodwell, 1996; Kim et al., 2000; Wilding et al., 2000). Beyond this well-known canonical action, statins have been found to induce direct alterations to lipid bilayer. Particularly, these alterations are marked by an increase in the elasticity of the membrane, independent of any changes in membrane cholesterol levels. Among the several statins tested, specifically fluvastatin emerged as the most active, similar to what was observed in our study (Sanford et al., 2013).

Although further studies are required to determine how statins have a direct effect on microbial viability, the importance of cholesterol for mycobacterial pathogenicity is undeniable. It has been reported that individuals with high dietary cholesterol have an increased risk of active tuberculosis (Soh et al., 2016). Likewise, Martens et al. observed that increased cholesterol levels in mice infected with *Mycobacterium tuberculosis* correlated with a greater bacterial load and accentuated pulmonary pathology (Martens et al., 2008). Regarding the possible association of Buruli Ulcer and levels of cholesterol, a recent metabolomic profiling in serum of Buruli Ulcer patients revealed a relative increase in circulating cholesterol and glucocorticoid hormones, when compared to healthy controls (Niang et al., 2015). The importance of cholesterol during mycobacterial infection is further highlighted when considering the reports on the effects of statin administration. Parihar et al. reported that *M. tuberculosis* growth was significantly reduced in human mononuclear cells and macrophages isolated from patients with familial hypercholesterolemia undergoing statin therapy, when compared with healthy donors (Parihar et al., 2014). Moreover, an epidemiological study showed that the cumulative use of statins and antibiotics was associated with a lower probability of developing pulmonary tuberculosis (Liao et al., 2017), and a meta-analysis underlined that statins may reduce the risk of active tuberculosis; however, heterogeneity and quality of evidence make any interpretation cautious (Meregildo-Rodriguez et al., 2022).

Our understanding of cholesterol’s role in *M. ulcerans* infection remains limited. Nevertheless, we do know that cholesterol-rich membranes have been described to facilitate mycolactone insertion into host cell membrane. This increased diffusion of the toxin through the membrane disturbs lipid organization, impairing the formation of ordered microdomains, crucial for normal cell function and signaling (Nitenberg et al., 2018). It remains currently unclear whether the cholesterol-lowering effect of statins could decrease mycolactone passive diffusion by preventing membrane remodeling. Cholesterol also contributes to the pool of metabolic building blocks, including acetyl-CoA, propionyl-CoA, succinyl-CoA, and pyruvate (Yam et al., 2009; Yang et al., 2009; Hu et al., 2010; Nesbitt et al., 2010; VanderVen et al., 2015), that are essential for mycolactone synthesis (Stinear et al., 2007; Robbe-Saule et al., 2021), raising the hypothesis that statins could affect toxin production. Moreover, these cholesterol-derived substrates accumulate within the caseous necrotic center of tuberculous granulomata, creating a permissive fatty acid-rich environment for *M. tuberculosis* persistence (Brzostek et al., 2009; Caceres et al., 2009; Ehrt and Rhee, 2013; Lovewell et al., 2016). The rupture of these necrotic structures releases mycobacteria into the respiratory tract, facilitating bacterial transmission. It would be interesting to determine if the necrotic acellular areas in the subcutaneous tissue of Buruli Ulcer patients could also act as a lipid-rich reservoir for the extracellular survival and persistence of *M. ulcerans*, while also facilitating transmission. Cholesterol has also been shown to impact host immunity. Specifically, statins have an effect on the cholesterol found within the host cell membrane hampering mycobacterial internalization (Gatfield and Pieters, 2000; Loike et al., 2004); statins override mycobacteria-induced arrest of phagosome maturation and promote lysosomal degradation (Gatfield and Pieters, 2000), and, statins modulate cytokine responses by promoting the release of pro-inflammatory cytokines IL-1β, IL-18 and IFN-γ (Montero et al., 2000). As outlined above, statins can have a number of pleiotropic effects, however which specific effects play a role in *M. ulcerans* infection warrants further investigation.

The most limiting factor for the implementation of statins as potential antimicrobial agents on their own is the concentration required to show antimicrobial effect (Gupta and Kumar, 2019), which is much higher than the therapeutic concentration found in the plasma of patients receiving cholesterol-lowering drugs (Bjorkhem-Bergman et al., 2011). Therefore, the use of statins for antimicrobial purposes would necessarily be carried out in combination with existing standard antimicrobial agents. Substantiating this potential, there is already existing evidence from both *in vitro* and *in vivo* studies showing statins capacity to enhance the efficacy of antibiotics and even to shorten the time of antibiotic action (Lobato et al., 2014; Skerry et al., 2014; Dutta et al., 2020). In the present work, we also tested the combination of atorvastatin and fluvastatin with the standard antibiotics used for the treatment of Buruli Ulcer. Although we did not see a synergistic effect between statins and antibiotics, the inclusion of statins to the antibiotic regimen showed an additive effect. Given the potential use of statins and antibiotics concomitantly, dose adjustment should be taken into account in future studies.

Drug repurposing is a practical and useful approach to exploit available and proven drugs outside the scope of the original medical indication. The result of this study highlights statins, a lipid-lowering drug, as a possible candidate for the adjuvant treatment of Buruli Ulcer. Beyond the antimicrobial potential of statins against *M. ulcerans*, the easiness of implementation in limited resource countries could be a convenient and timely strategy to maximize assets.

## Data availability statement

The raw data supporting the conclusions of this article will be made available by the authors, without undue reservation.

## Ethics statement

Ethical approval was not required for the studies on humans in accordance with the local legislation and institutional requirements because only commercially available established cell lines were used.

## Author contributions

JD: Conceptualization, Writing – original draft, Writing – review & editing, Formal analysis, Investigation, Methodology, Visualization. AM: Formal analysis, Investigation, Methodology, Writing – review & editing. AP: Formal analysis, Investigation, Writing – review & editing, Validation. MP: Formal analysis, Investigation, Writing – review & editing. JP: Writing – review & editing, Conceptualization, Funding acquisition, Resources, Supervision, Writing – original draft. AF: Conceptualization, Funding acquisition, Resources, Supervision, Writing – original draft, Writing – review & editing.

## Funding

The author(s) declare financial support was received for the research, authorship, and/or publication of this article. This work has been funded by National funds, through the Foundation for Science and Technology (FCT) – project UIDB/50026/2020, UIDP/50026/2020, and 2022.05266.PTDC and by the projects, NORTE-01–0145-FEDER-000039 and NORTE-01–0145-FEDER- 085468, supported by Norte Portugal Regional Operational Program (NORTE 2020), under the PORTUGAL 2020 Partnership Agreement, through the European Regional Development Fund (ERDF). JD would like to acknowledge the individual grant from SENESCYT scholarship.

## Conflict of interest

The authors declare that the research was conducted in the absence of any commercial or financial relationships that could be construed as a potential conflict of interest.

## Publisher’s note

All claims expressed in this article are solely those of the authors and do not necessarily represent those of their affiliated organizations, or those of the publisher, the editors and the reviewers. Any product that may be evaluated in this article, or claim that may be made by its manufacturer, is not guaranteed or endorsed by the publisher.
