# Supplementary material for: Repurposing of statins for Buruli Ulcer treatment: antimicrobial activity against Mycobacterium ulcerans
Source: Front Microbiol. 2023 Sep 29;14:1266261. doi: 10.3389/fmicb.2023.1266261 (PMC10570734; doi:10.3389/fmicb.2023.1266261)
Supplement: Supplementary file 1 [file Data_Sheet_1.PDF]

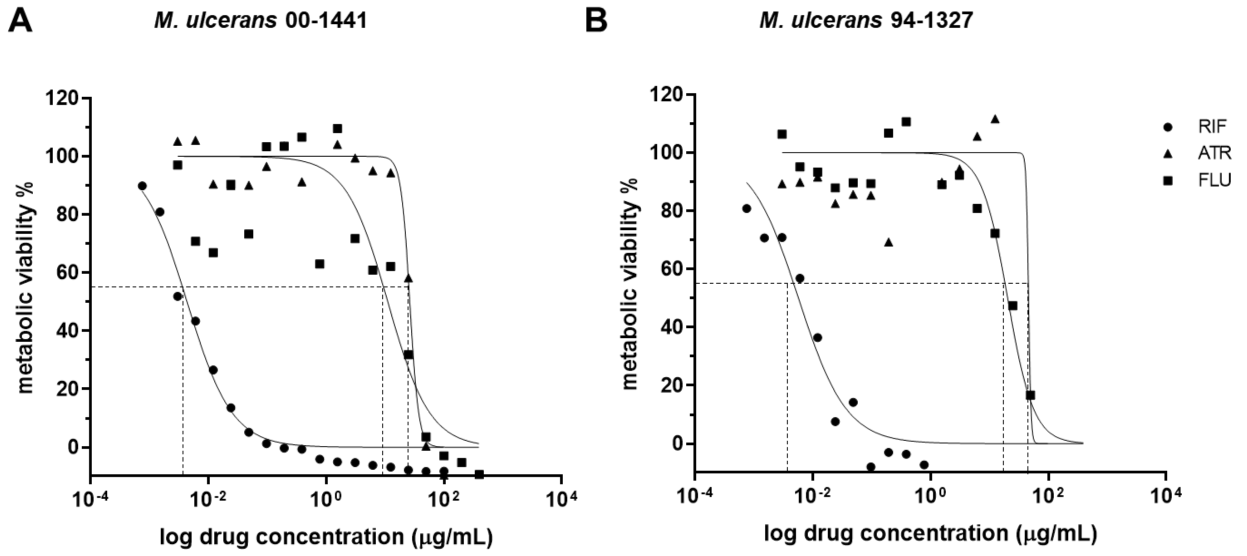

Supplementary Figure S1. Dose-response curves for the antimicrobial activity of statins against *M. ulcerans.* The broth microdilution assay was used to determine the dose-response curves of **(A)** *M. ulcerans* 00-1441 and **(B)** *M. ulcerans* 94-1327 against statins. The different strains of *M. ulcerans* were incubated for 7 days with increasing concentrations of statins [0.003052 - 400 μg/mL] (ATR – atorvastatin; FLU – fluvastatin) or with the standard antibiotic for Buruli Ulcer treatment [0.000763 - 100 μg/mL] (RIF – rifampicin). After this period of incubation, metabolic viability of *M. ulcerans* was determined with resazurin. Dashed lines represent the IC50 for each statin. Two independent experiments are represented.
